# Detection of Manipulated Face Videos over Social Networks: A Large-Scale Study

**DOI:** 10.3390/jimaging7100193

**Published:** 2021-09-28

**Authors:** Federico Marcon, Cecilia Pasquini, Giulia Boato

**Affiliations:** Department of Information Engineering and Computer Science, Via Sommarive 9, 38123 Trento, Italy; federico.marcon@alumni.unitn.it (F.M.); giulia.boato@unitn.it (G.B.)

**Keywords:** deepfakes, video forensics, facial manipulations, social networks, deep learning

## Abstract

The detection of manipulated videos represents a highly relevant problem in multimedia forensics, which has been widely investigated in the last years. However, a common trait of published studies is the fact that the forensic analysis is typically applied on data prior to their potential dissemination over the web. This work addresses the challenging scenario where manipulated videos are first shared through social media platforms and then are subject to the forensic analysis. In this context, a large scale performance evaluation has been carried out involving general purpose deep networks and state-of-the-art manipulated data, and studying different effects. Results confirm that a performance drop is observed in every case when unseen shared data are tested by networks trained on non-shared data; however, fine-tuning operations can mitigate this problem. Also, we show that the output of differently trained networks can carry useful forensic information for the identification of the specific technique used for visual manipulation, both for shared and non-shared data.

## 1. Introduction

Latest advancements in artificial photo-realistic generation enabled new outstanding possibilities for media data manipulations. So-called *deepfakes*, i.e., credible digital media depicting untruthful content, can be obtained either through the manipulation of pristine material or generated from scratch thanks to automated algorithms based on Artificial Intelligence (AI). The web abounds with tutorials and applications for the creation of simple deepfakes products that can easily run on a commercial smartphone or PCs (such as FakeApp, Impressions, Reface App, MyVoiceyourface, Snapchat Cameos, FaceSwap), and more sophisticated creation techniques are developed at a fast pace.

These technologies poses significant threats to the reliability of visual information, and can represent harmful tools to undermine the digital identity and reputation of individuals. The many cases of abuses reported in the last months involving public figures in politics and economics, confirm these concerns, and we can only expect this phenomenon to increase in the upcoming years. As a response, the detection of the employment of new efficient techniques for synthetic media generation has drawn many research efforts in the last years [1]. An ever increasing number of tools and approaches have been proposed in the last years, together with the development of benchmark datasets (e.g., FaceForensics++ [2]) and world-wide open challenges (e.g., Facebook Deepfake Detection Challenge).

While earlier approaches were focused on the detection of imperfections, artifacts, distortions in the outcomes, the recent success of deep learning for visual analysis brought researchers to employ also purely data-driven detection methodologies. Indeed, general purpose neural networks have shown encouraging results in detecting video frames that have been manipulated [2,3].

While several methodologies and datasets have been published during the last years, one rather unexplored aspect is the generalization capability of those deep descriptors in situations where data are shared through social platforms [3,4]. This is a known and ever emerging problem in multimedia forensics [5], given the pervasive role of popular social media platforms in the dissemination and exchange of visual content on a daily basis.

In this regard, this work presents the results of an extensive detection analysis which goes beyond controlled laboratory conditions, typically adopted in previous works [1], and deals with a scenario where data are not only analyzed as direct outputs of manipulation algorithms but also after upload/download operations through a popular sharing service. As it has been observed in previous studies [6,7], the uploading/downloading steps involved in the sharing process typically operate heavily on the data under investigation, for instance through resizing and recompression to save memory and bandwidth. Thus, while non-shared data better exhibit the inter-pixel statistics at the center of feature-based extraction and analysis, such sharing operations impacts pixel distribution and potentially compromise the detection capabilities of forensics detectors.

While the addressed scenario is of high practical relevance due to the massive daily use of social media platforms for content dissemination, extensive experimental studies in this context are hindered by the high workload required in the data collection phase. In fact, the upload/download operations through different platforms can rarely be automated efficiently and are typically performed in a semi-manual fashion.

We can then summarize the contributions of this work as follows:

- we created an enlarged data collection of shared manipulated videos that is available to the scientific community (Data can be downloaded at: https://tinyurl.com/puusfcke, accessed on 17 September 2021);
- we provide empirical evidences of generalization and transfer learning capabilities of CNN-based detectors;
- we devise and evaluate a simple ensemble strategy to trace the specific manipulation algorithm of data that are detected as fake.

The remainder of the paper is structured as follows: in Section 2, we provide an overview of previous works addressing the discrimination between synthetic and real faces, focusing in particular on manipulated video sequences. In Section 3, we presents our experimental design and setting with the involved deep architectures, data and sharing platforms. In Section 4, we describe the results emerging from our experimental campaign on pre-social and post-social videos, also with respect to the ability of identifying the manipulation technique and performing a video-based decision. Finally, in Section 5 we draw some conclusive remarks.

## 2. Related Work

In this section, we recall the main approaches employed in the literature for the detection of manipulated facial data. Due to the abundance of techniques proposed in the recent years, we outline here a general categorization and group the different approaches according to their main rationale, while referring the interested reader to [1] for a detailed review.

### 2.1. Methods Based on Physical Inconsistencies

The first generation of deepfakes contents used to exhibit visible visual inconsistencies in generating human faces and expressions. For this reason, the research was initially directed at detecting, for instance, miss-matching eye blinking [8], as the manipulation algorithms, being trained on images showing people with open eye, were unable to realistically reproduce this phenomena. However, creation technologies have been constantly improving and reducing those artefacts, as it is shown in [9].

The work proposed in [10] exploits the limitations of AI in producing faces at fixed size, and adapted through affine transformation to different target poses, by training a CNN on “good” and “bad” fake examples to recover the warping artifacts.

Similarly, the strategy proposed in [11] is focused on alignment errors of synthesized faces in non-frontal head poses or critical situations such as rapid changes in illumination or distance from camera. Moreover, detection approaches operate on the basis of color disparities [12] since fake media, being usually generated using only on RGB images, exhibit substantial differences in other color spaces with respect to real contents that, through acquisition process, are subjected to specific relation in their color components.

### 2.2. Methods Based on Handcrafted Descriptors

Earlier studies perform classification between real and manipulated content focusing on statistical features related to specific traces of real data during acquisition process [13], such as color filter array interpolation [14] and lens chromatic aberration [15].

A detection based on handcrafted feature starting from noise residual [16] from videos in FaceForensics++ are used to train a SVM classifier with good performance, but only without compression present. The process of residual-based forgery detection is also implemented through CNN architecture in [17]. In this context, other approaches include the analysis of the spatio-temporal texture [18,19,20] and of distributions of coefficients in wavelet domain [21,22]. Moreover, differently from common approaches where the analysis is usually performed in the image domain, [23] examined GAN-generated images in the frequency domain demonstrating how artifacts can be recovered with this representation.

### 2.3. Methods Based on Biological Signals Extraction

Along with the idea to develop fake detection on the natural characteristics or behaviors of human beings, several works have been presented [24]. DeepRhythm [25] classifies real or computer generated faces exploiting heart rate (HR) manifestations in the periodic color skin variations caused by the flowing of blood. FakeCatcher [26] has been designed building on photoplethysmography, the optical technique used to detect volume variation of blood flowing, thanks to its robustness against dynamic changing of the scene. The aim of DeepPhyON [27] is to adapt the features learned for HR estimation with DeepPhys [28], a model designed to isolate the information of color changes caused by fluctuations of oxygen level in blood from the one related to other factor like illumination and noise conditions.

In this context, another promising stream of research analyzes the facial spatio-temporal dynamics by tracking face landmarks over time and building soft biometrics models of individuals [29,30].

### 2.4. Methods Based on Deep Descriptors

In light of the success of deep learning in many close fields, researchers have extensively applied Convolutional Neural Networks (CNN) as manipulation detectors [31], due to their ability to automatically learn the more relevant descriptors.

In [32], a CNN-based analysis is performed for the distinction between real and computer-generated images by combining the contribution of small patches under the same image. Inspired by the Inception architecture [33], mesoscopic features are employed in [34]. In general, the work in [2] shows that deeper general-purpose networks like Xception largely outperform shallow ones such as [32,34,35], as well as re-adapted feature-based methods originating from steganalysis [16]. One of the most recent studies [36] addresses the problem of identifying and locating fake faces when more than one are present in the same scene. After creating a new large scale dataset, the authors implemented the detection based on CNNs to obtain an algorithm that could be more robust when varying the number of targets in a video and that could automatically learn where the manipulation occured.

The majority of proposed studies are based on benchmark datasets, but rarely consider the scenario where data undergo further post-processing after the manipulation process. In particular, the impact of sharing operations on social networks, routinely performed to acquired data, is largely unexplored and, to the best of our knowledge, the only contribution in this regard can be found in [4]. However, such work operates on data where the upload/download operation is only simulated through hard-coded compression and no actual sharing through existing and active platforms is performed.

## 3. Experimental Design and Settings

**Figure 1:**
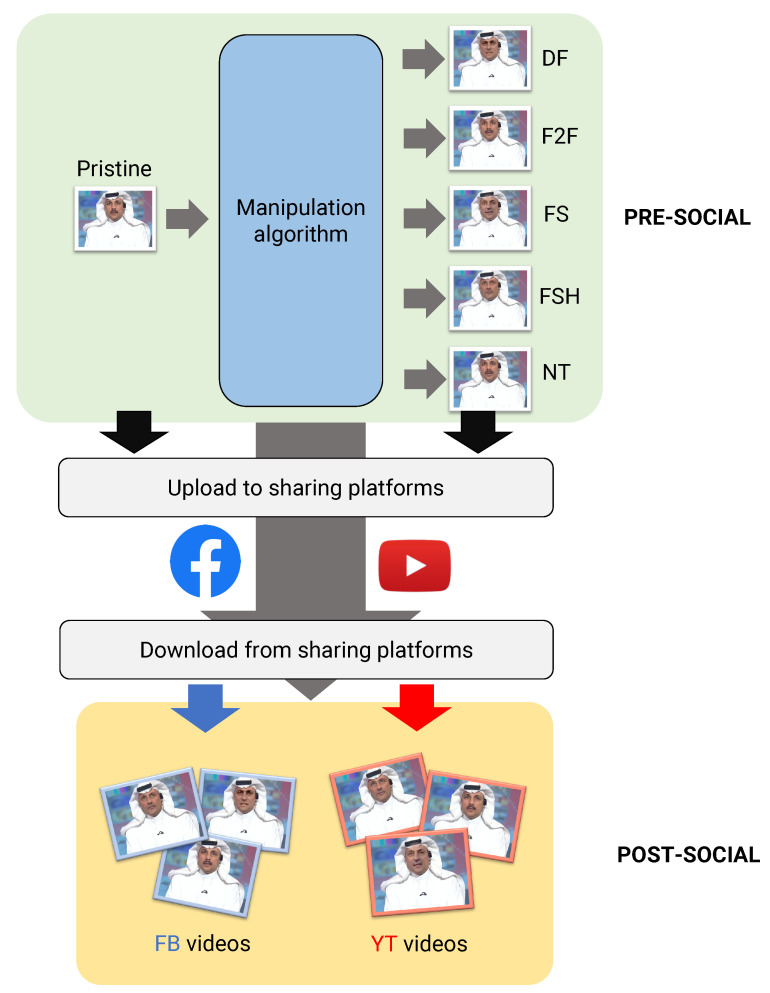
Experimental design of our comparative evaluation.

We now outline the design of our empirical analysis and describe the experimental settings considered. The overall framework is depicted in Figure 1, where the *pre-social* and *post-social* scenario are represented.

In the first case, data are analyzed as direct outputs of the manipulation operations, followed only by a high-quality compression. In the second case, data are uploaded and downloaded through social networks.

### 3.1. Initial Data Corpus

In order to carry out our quantitative experiments, we build on the state-of-the-art dataset FaceForensics++ [2], created under the necessity of providing the community with a large-scale video dataset for face manipulation analysis.

FaceForensics++ consists of 1000 original videos, each of them manipulated through 5 different manipulation techniques Deepfake (DF), Face2Face (F2F), FaceSwap (FS), NeuralTextures (NT) and FaceShifter (FSH) techniques. All the videos depict one person, typically in a central position within the frame. In terms of manipulation type, Face2Face and NeuralTextures perform video re-enactment, thus the facial dynamics of a source video is transferred into a target video. The Deepfake, FaceShifter and FaceSwap technique instead implement face substitution, thus superimposing the face taken from a source video into the facial dynamics of a target video.

Also, different techniques present diversity in terms of tools they employ: Deepfakes, NeuralTextures, FaceShifter are based on pre-trained learning-based schemes, while FaceSwap and Face2Face rely on computer graphics rule-based methodologies. FaceForensics++ offers three different quality versions: raw (unprocessed video frames), high-quality (HQ) with 23 factor compression and low-quality (LQ) 40-compressed. While raw video frames are unlikely to be encountered, LQ videos are strongly degraded; thus, we focus our analysis on HQ compressed videos as a tradeoff between visual quality and practical relevance.

The dataset comes with a predefined partition of the 1000 videos into training, validation and testing set (composed by 720, 140 and 140 videos, respectively), which we also employed in our experiments.

### 3.2. Deep Architectures for Detection

For comparison purposes, we consider three different general purpose CNNs that proved to be effective in the classification of real and manipulated contents:

- *Xception* (XC) [37] was born as “extreme” version of Inception architecture and it is proved in [36,38,39] as proficient backbone architecture for forensic detectors (in [2] it is reported as the most successful architecture on the FaceForensics++ dataset).
- *InceptionV3* (INC) [40] is the result of improvements to the original Inception structure [33] and based on multiple filters of different sizes in the same module to enhance scalability of descriptors. It has been used in image forensics for copy-move forgery detection [41] and GAN-generated image detection [3].
- *Densenet* (DEN) [42] is designed on dense connections ensuring large diversity of features with few parameters. It has found applications in image classification [43], steganalysis [44], and the identification of GAN-generated images shared on social networks [3].

All of them operate in a frame-wise fashion, thus the analysis is performed on single frames without considering the temporal relation between them. In each training phase, we reproduce the procedure adopted in [2]: starting from models pretrained on Imagenet, the classification layer is separately pretrained for 3 epochs, and then the full network is trained for 15 epochs and the model with best validation accuracy is chosen. Regarding training hyperparameters, samples are grouped in batches of 32 and Adam optimizer is applied with its default values and learning rate equal to 0.0002.

### 3.3. Data Creation

Both pristine and manipulated videos have been uploaded to and downloaded from two popular platforms, YouTube (YT) and Facebook (FB). Such operations have been performed in a semi-manual fashion for each video in the validation and testing set and for each manipulated version, leading to a total number of shared videos equal to (140+140)×6×2=3360.

In particular, on YouTube the procedure is managed through the YouTube Studio interface where video playlists can be created with a maximum of 15 videos uploaded per day. Successively, each sequence is downloaded individually from the playlist. For the case of Facebook, since no constraints on the number of videos are in place, videos have been published as private albums; the downloading operation is applied in batch through the “Download album” functionality.

The degradation of the videos, once shared, is confirmed when observing the downscaling in resolution and the decrease in size of files. In terms of resolution, pre-social videos undergo a reduction of an average factor of 0.8 on Facebook and 0.64 on YouTube. Similarly, the file dimension is respectively impacted of 0.5 and 0.7 after the downloading.

## 4. Experimental Analysis

We now report the main results of our evaluation campaign.

In our analysis, the three architectures are always trained individually to distinguish real from manipulated frames for each single technique of FaceForensics++. In order to conduct extensive comparative tests, a set of *baseline* binary detectors have been trained by employing the 3 different architectures and the 5 different manipulation techniques. This leads to 15 baseline models indicated as ${\mathrm{XC}}_{m}$, ${\mathrm{INC}}_{m}$ and ${\mathrm{DEN}}_{m}$, where m∈{DF,F2F,FS,FSH,NT}.

By following the same settings as in [2], the binary video classification is always performed at frame level (unless otherwise stated) by extracting 10 frames from each video. In doing so, a face detector is applied to identify the face area, which is then cropped and fitted to the input size of the networks. Thus, according to the data splits provided, for every detector we have 7200 training frames, 1400 validation frames and 1400 testing frames for each class.

The remainder of the section is structured as follows:

- *Detection Performance in the Pre-Social Scenario (Section 4.1)*
  videos are first analyzed in their pre-social version, showing consistent results with what reported in [2];
- *Generalization Performance in the Post-Social Scenario (Section 4.2)*
  the analysis is extended to shared data and the performance of deep networks is evaluated in a standard and transfer learning mode;
- *Identification of the Manipulation Technique (Section 4.3)*
  we evaluate the possibility of identifying the manipulation technique that has been used to create the video by exploiting the different network outputs;
- *Accuracy of Video-based Aggregated Decisions (Section 4.4)*
  the analysis of individual frame is combined to obtain a decision on the full video.

### 4.1. Detection Performance in the Pre-Social Scenario

**Figure 2:**
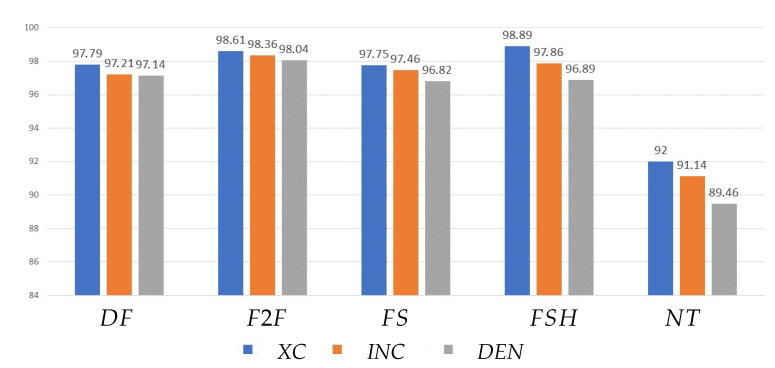
Accuracy of different networks in the pre-social scenario.

The chart in Figure 2 reports the accuracy results for all the deep networks when distinguishing pristine and manipulated video frames extracted from videos in the pre-social scenario. Results are reported separately for each manipulation technique and show in general good discrimination capabilities.

Among the considered nets, Xception (XC) always provides superior performance against every manipulation, in line with what obtained in [2] when comparing Xception to other forensic detectors. However, InceptionV3 (INC) and Densenet (DEN) also exhibit rather high accuracy, with a maximum decrease with respect to Xception equal to 1.0% and 2.54%, respectively.

When observing the results across different manipulations, we can moreover observe that the detection accuracy on data manipulated through NT is significantly lower (no higher than 92.0%), while for all the other four techniques we achieve an accuracy above 96% in all cases.

### 4.2. Generalization Performance in the Post-Social Scenario

We now report the results of the post-social analysis, which include measuring different effects, as described below.

First, we directly test the baseline models already trained in Section 4.1 on video frames extracted from shared videos. This first allows us to measure what we indicate as the *misalignment loss*, defined as the decrease in accuracy observed for the baseline models when moving from tests on pre-social data to tests on post-social data.

Then, we evaluate the effectiveness of a simple transfer learning strategy via finetuning. In particular, pretrained baseline models are further trained on a number of frames extracted from shared videos. For this purpose, we used the videos in the validation set (140 for each binary class). We applied this procedure for every baseline model and both sharing platforms, leading to 30 so-called *specialized* models. Thus, each of them is first trained on real and manipulated frames created to a specific manipulation and then fine-tuned with real and manipulated frames of videos shared from a given platform. We indicate as subscript the platform on which detectors are specialized, so that, for instance, ${\mathrm{XC}}_{m}^{YT}$ is the specialized model obtained by fine-tuning ${\mathrm{XC}}_{m}$ with validation data shared through YouTube.

By doing so, we can then evaluate two other effects, namely:

- the *fine-tuning gain*, defined as the increase in accuracy observed on post-social data when specialized models are employed in place of baseline models;
- the *forgetting loss*, the decrease in accuracy observed on pre-social data when specialized models are employed in place of baseline models. (The terms “loss” and “gain” are used by definition to indicate a decrease and an increase in accuracy, respectively, due to direction of the expected effect. They might however assume negative values, thus indicating a reversed effect (e.g., a negative loss indicates an increase in accuracy)).

In fact, in addition to measuring the advantages of using specialized detectors on the newly seen post-social data, it is also important to evaluate to which extent they remain accurate on pre-social data, for which they had been originally trained.

In order to effectively visualize those observed effects, we report the results of the different tests in a condensed format by adopting in the plots the following convention:

△ accuracy of baseline models on pre-social data
○ accuracy of baseline models on post-social data
● accuracy of specialized models on post-social data
▲ accuracy of specialized models on pre-social data

**Figure 3:**
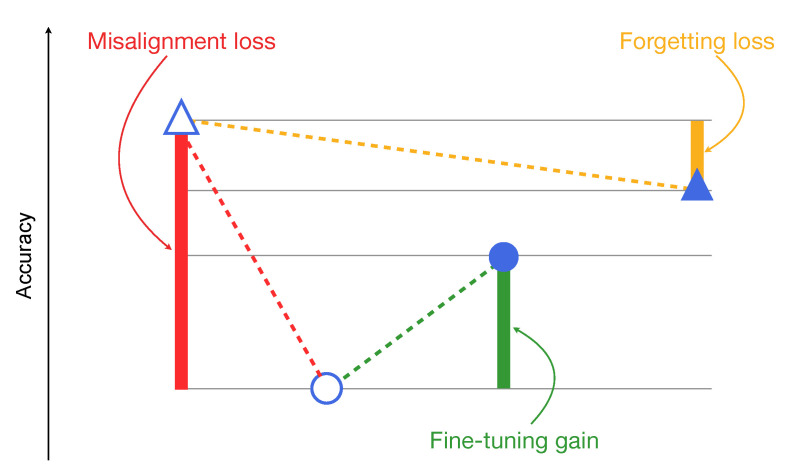
Example of result visualization.

**Figure 4:**
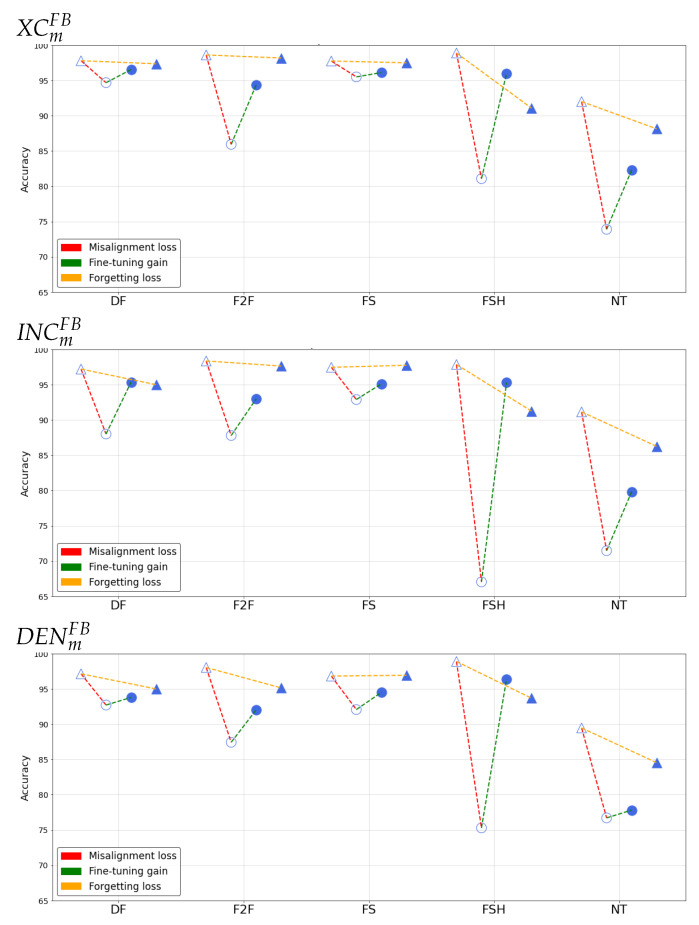
Accuracy results in the post-social scenario on FB.

**Figure 5:**
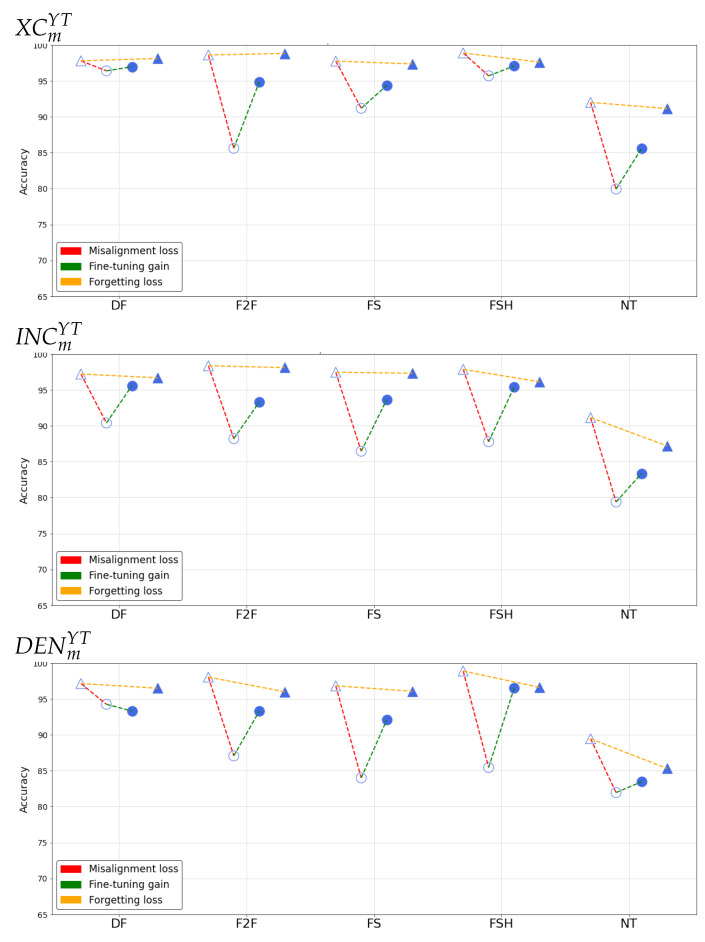
Accuracy results in the post-social results on YT.

By doing so, in each case we can represent the results as depicted in Figure 3:

Figure 4 and Figure 5 report the results of such analysis for the different networks, manipulation techniques and sharing platforms.

We can then observe the different effects separately:

**Misalignment loss**

The extent of the loss varies across the manipulations considered, the employed architecture and the sharing platform. In general, we can deduce that XC is on average the most robust in detecting manipulated content in presence of strong degradation of information, while Densenet seems to be relatively less susceptible against NT. By looking at different manipulation techniques, the two platforms seem to have different impact: for FB, FSH and NT are the ones resulting more challenging to detect, while for YT higher misalignment losses are observed also on F2F and FS. In general, the loss is particularly small for DF data.

**Fine-tuning gain**

When fine-tuning is applied to post-social data, the gain is always positive and sometimes reaches 20%. Accuracy is brought above 90% in every case, except for NT data for which the detection capabilities are strongly compromised in the post-social scenario. The only exception is given by the ${\mathrm{DEN}}_{DF}^{YT}$ model, which slightly decreases the performance of ${\mathrm{DEN}}_{DF}$ on post-social data. This confirms the peculiar behaviour of the DF manipulated data with respect to the other techniques.

For the sake of completeness, we report in Table 1 and Table 2 the full accuracy results obtained through specialized models.

**Forgetting loss**

By looking at the forgetting loss, we can notice that its behaviour varies considerably among different manipulation techniques, showing essentially small performance fluctuations on not-shared content when adopting baseline and specialized detectors for the DF, F2F and FS techniques. For FSH and NT, the forgetting loss increases, mostly in correspondence to higher values of the misalignment loss.

### 4.3. Identification of the Manipulation Technique

Although less investigated with respect to the distinction between real and manipulated content, one interesting aspect in this experimental framework would be the ability to blindly identify the manipulation technique used for altering the video. In fact, in a video verification scenario, determining which algorithmic pipeline has been employed on data that have been reported as manipulated could aid the process of tracing users or services which provided the untruthful visual content [5].

Therefore, we address this problem and explore the possibility of exploiting for this purpose the outputs of our different binary networks. In fact, predictions on single frames made by the considered deep networks come in the form of a value in [0,1] (the softmax layer output), which is interpreted as the probability of the sample to belong to the manipulated class and successively binarized. Thus, if is x a generic frame and *F* as a generic model, we can indicate as F(x)∈[0,1] the model output; when F(x)>0.5, the x is classified as manipulated.

In each configuration, both our baseline and specialized models are exposed during training to manipulated data created with only a certain technique; we can then expect that the network predictions will be higher when manipulated frames produced through this specific technique are tested, with respect to other kinds of frames.

For a generic testing frame x and the tree architectures considered, we then define the sets

(1) $$\begin{array}{cc} \mathrm{XC}(x) & =\{{\mathrm{XC}}_{DF}\left(x\right),{\mathrm{XC}}_{F2F}\left(x\right),{\mathrm{XC}}_{FS}\left(x\right),{\mathrm{XC}}_{FSH}\left(x\right),{\mathrm{XC}}_{NT}\left(x\right)\} \end{array}$$

(2) $$\begin{array}{cc} \mathrm{INC}(x) & =\{{\mathrm{INC}}_{DF}\left(x\right),{\mathrm{INC}}_{F2F}\left(x\right),{\mathrm{INC}}_{FS}\left(x\right),{\mathrm{INC}}_{FSH}\left(x\right),{\mathrm{INC}}_{NT}\left(x\right)\} \end{array}$$

(3) $$\begin{array}{cc} \mathrm{DEN}(x) & =\{{\mathrm{DEN}}_{DF}\left(x\right),{\mathrm{DEN}}_{F2F}\left(x\right),{\mathrm{DEN}}_{FS}\left(x\right),{\mathrm{DEN}}_{FSH}\left(x\right),{\mathrm{DEN}}_{NT}\left(x\right)\}. \end{array}$$

Analogous sets can be defined in the same way when specialized models are used by simply adding the corresponding superscript.

Building on this rationale, one can conjecture that the maximum response observed among the five different available deep detectors can act as an indicator of the manipulation technique on a generic frame. Then, we blindly analyze each testing frame x and provide three estimates of the manipulation technique as the ones corresponding to maxXC(x), maxINC(x) and maxDEN(x).

**Figure 6:**
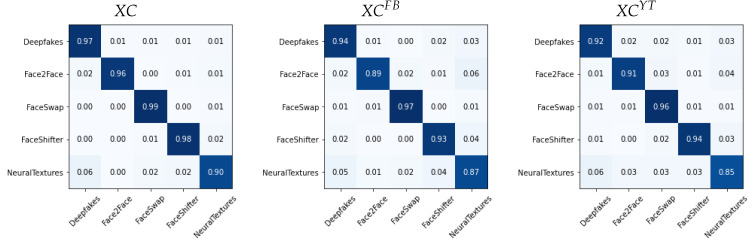
Confusion matrices obtained from XC(x) in the pre-social and post-social scenarios.

**Figure 7:**
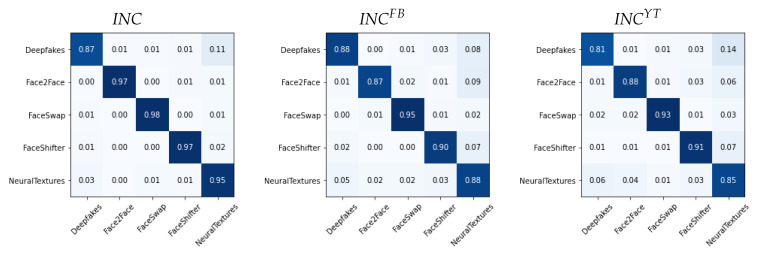
Confusion matrices obtained from INC(x) in the pre-social and post-social scenarios.

**Figure 8:**
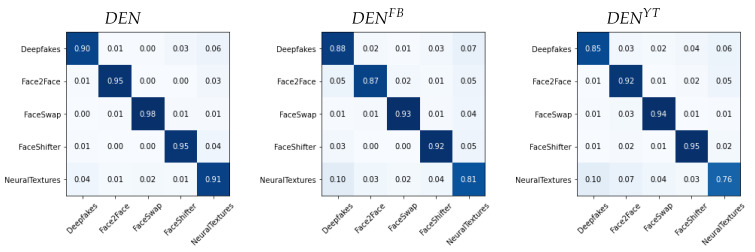
Confusion matrices obtained from DEN(x) in the pre-social and post-social scenarios.

We report in Figure 6, Figure 7 and Figure 8 the confusion matrices obtained with such methodology for the different architectures. In each case, we tested both the pre-social and the post-social scenarios, the latter being addressed with specialized models.

We observe a clear diagonal in each case, with comparable performance when comparing the pre- and post-social scenarios, thus demonstrating that the network outputs indeed carry useful forensic information for this task. When observing the behaviour of specific manipulation techniques, we notice that Deepfakes (DF) and NeuralTextures (NT) consistently present a higher error.

### 4.4. Accuracy of Video-Based Aggregated Decisions

While the analyzed models perform a forensic analysis on individual frames (by extracting 10 frames per video), in practical situations those predictions are typically combined in order to take a decision on the entire multimedia object, i.e., the full video.

Thus, we here evaluate the ability of frame-wise decisions (based on deep network predictions) to support a video-wise decision. In particular, instead of selecting a limited number of frames per video, we now analyze all of them through the nets.

For each full video, we compute all the binary responses of individual frames and the ratio of frames that are classified as manipulated.

Such ratio value can be thresholded in order to take a decision on each video, so that a Receiver Operating Curve can be produced for a varying threshold t∈[0,1]. When t=0.5, the decision rule corresponds to a majority voting criterion over multiple frames. False and true positive rate are here computed on the total number of test videos.

**Figure 9:**
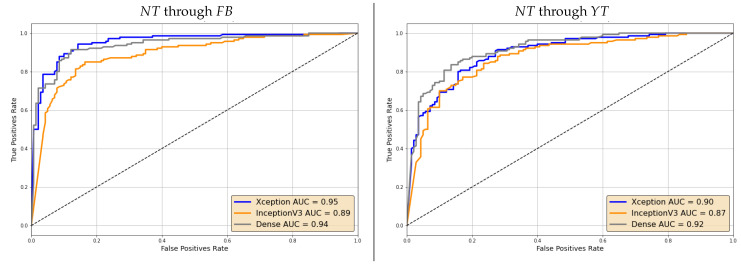
ROC curves of the video-based decision through specialized models on NT videos shared on Facebook (**left**) and YouTube (**right**).

**Figure 10:**
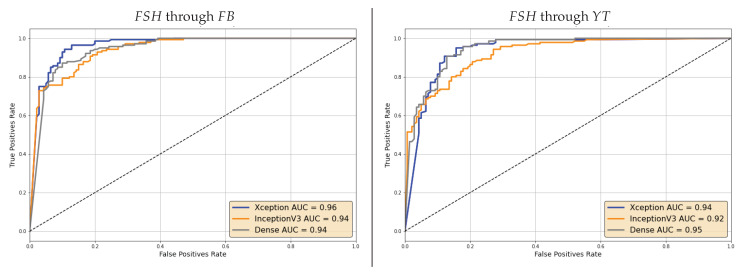
ROC curves of the video-based decision through specialized models on FSH videos shared on Facebook (**left**) and YouTube (**right**).

For the sake of brevity, we limit this analysis to FSH and NT analyzed through specialized models in the post-social scenario. The resulting ROCs are reported in Figure 9 and Figure 10. We can notice that Area Under the Curve (AUC) values are rather high in all cases, thus showing that lower accuracy values on individual frames can indeed be mitigated by the aggregation of multiple ones.

In general, the discrimination capability seems however to decrease when videos are shared through YT. This holds for both the selected manipulations.

## 5. Conclusions

In this work we have addressed the challenging scenario where forensics analysis is applied to manipulated videos shared through social media platforms.

Indeed, we have presented an extensive evaluation going beyond controlled laboratory conditions and analyzing detection performance both in what we have called pre-social and post-social scenarios, involving several general purpose deep networks, state-of-the-art manipulated data and two popular sharing platforms (Facebook and YouTube).

We have shown generalization and transfer learning capabilities of CNN-based detectors measuring misalignment loss, fine-tuning gain and forgetting loss for all different types of data and architecture. Moreover, we have presented an ensemble strategy to identify the specific manipulation algorithm of data that are detected as fake. Finally we have analyzed detection performance when moving from single frame prediction to full video sequence decision, where predictions on every frame are aggregated and the decision between real and fake is given by the percentage of fake frames identified.

All such results show promising directions for an effective forensic analysis in real-world scenarios where deceptive media are shared after manipulation. In particular, simple transfer learning via fine-tuning seems a viable strategy for re-gaining accuracy when the testing data deviates from the training one due to the sharing operation. In this regard, alternative and possibly more efficient data augmentation techniques could be explored that simulate the various resizing and recompression pipelines of social networks, which are however not fully known. In this framework, issues can be however foreseen due to the purely data-driven nature of this methodology. In fact, in our tests a limited number of platforms were selected and analyzed separately, always assuming some kind of knowledge on this regard. Dealing with a higher number of platforms in the training phase, as well as in blind scenarios where unseen platforms are involved in the testing phase represent open problems for future investigations. Related to this, provenance studies could also be performed with the goal of identifying the sharing pipeline of the analyzed content and thus facilitate the forensic analysis. Moreover, a possible way to overcome the need for extensive training data in the data-driven techniques would be to employ methods based on physiological cues or physical inconsistencies, whose robustness to sharing processes should be assessed. Finally, one open point which would deserve further investigation is the relation between the specific manipulation technique with respect to the detector performance.

## Figures and Tables

**Table 1:** Accuracy of networks fine tuned on Facebook videos.

	DF	F2F	FS	FSH	NT
XC	96.54	94.39	96.11	95.39	82.29
INC	95.36	93.00	95.11	95.29	79.82
DEN	93.79	92.04	94.50	95.36	77.82

**Table 2:** Accuracy of networks fine tuned on YouTube videos.

	DF	F2F	FS	FSH	NT
XC	96.96	94.86	94.36	97.07	85.57
INC	95.57	93.32	93.64	95.39	83.36
DEN	93.38	93.12	92.07	96.50	83.46

## Data Availability

The initial data corpus (FaceForensics++ data) is available at https://github.com/ondyari/FaceForensics, accessed on 17 September 2021.
